# Trait anxiety and coping strategies among healthcare professionals during the COVID-19 pandemic

**DOI:** 10.1192/j.eurpsy.2021.1783

**Published:** 2021-08-13

**Authors:** N. Smaoui, A. Bouaziz, M. Kraiem, S. Omri, R. Feki, M. Maalej Bouali, N. Charfi, J. Ben Thabet, L. Zouari, M. Maalej

**Affiliations:** 1 Psychiatry C Department, Hedi chaker University hospital, sfax, Tunisia; 2 Psychiatry C Departement, Hospital University of HEDI CHAKER, sfax, Tunisia; 3 Psychiatry C, Hedi Chaker University Hospital, sfax, Tunisia

**Keywords:** Healthcare professionals, Trait-Anxiety, Coping strategies, Covid-19 pandemic

## Abstract

**Introduction:**

The current pandemic wave of COVID-19 has become a global threat. Healthcare professionals (HCP), by being on the front line in managing this pandemic, confronted high levels of stress and traumatic experiences.

**Objectives:**

The aims of our study were to evaluate the trait-anxiety among Tunisian HCP and its impact on coping strategies.

**Methods:**

A cross- sectional descriptive and analytic study conducted among Tunisian HCP during November and December 2020. The data was collected by an online questionnaire distributed through social media. The trait-anxiety was assessed using the “General Anxiety questionnaire of Spielberger” (STAI-Y-B) and the “Ways of coping checklist revised (WCC-R) questionnaire” identified three types of coping (problem-focused, emotion-focused and social-support seeking).

**Results:**

Participants were 135 HCP (71 males and 64 females) and aged from 24 to 61 years old (average age 31.98 years; SD 6.59 years). Of HCP involved in the study, 61.5% were single, 36.3% were married and 2.2% were divorced. Seventy-two of them had a trait-anxious. As a coping style, 85.2% of participants used problem-focused style, 88.9% of them used emotion-focused style and 63% of them used social support seeking style. The analysis of WCC-R showed that anxious HCP used emotion-focused coping more than non anxious HCP (p= 0.028). However, there was no significant difference in problem-focused or social support seeking coping styles and presence of trait-anxiety.

**Conclusions:**

In our study, we find that the most anxious Tunisian HCP focused on emotions strategies. Personality traits play on attitudes of coping strategies.

**Disclosure:**

No significant relationships.

